# Eco-physiological responses of copepods and pteropods to ocean warming and acidification

**DOI:** 10.1038/s41598-019-41213-1

**Published:** 2019-03-18

**Authors:** J. Engström-Öst, O. Glippa, R. A. Feely, M. Kanerva, J. E. Keister, S. R. Alin, B. R. Carter, A. K. McLaskey, K. A. Vuori, N. Bednaršek

**Affiliations:** 1 Novia University of Applied Sciences, Ekenäs, Finland; 2 Pacific Marine Environmental Laboratory, National Oceanic and Atmospheric Administration, Seattle, WA USA; 3 Ehime University, Center for Marine Environmental Studies, Laboratory of Environmental Toxicology, Matsuyama, Japan; 4 School of Oceanography, University of Washington, Seattle, USA; 5 Joint Institute for the Study of the Atmosphere and Ocean, University of Washington, Seattle, USA; 6 Laboratory of Animal Physiology, Department of Biology, University of Turku, Turku, Finland; 7 Southern California Coastal Water Research Project, Costa Mesa, CA 92626 USA

## Abstract

We compare physiological responses of the crustacean copepod *Calanus pacificus* and pelagic pteropod mollusk *Limacina helicina* to ocean temperatures and pH by measuring biomarkers of oxidative stress, antioxidant defences, and the activity of the respiratory electron transport system in organisms collected on the 2016 West Coast Ocean Acidification cruise in the California Current System. Copepods and pteropods exhibited strong but divergent responses in the same habitat; copepods had higher oxygen-reactive absorbance capacity, glutathione-S-transferase, and total glutathione content. The ratio between reduced to oxidised glutathione was higher in copepods than in pteropods, indicating lower oxidative stress in copepods. Pteropods showed higher activities of glutathione reductase, catalase, and lipid peroxidation, indicating increased antioxidant defences and oxidative stress. Thus, the antioxidant defence system of the copepods has a greater capacity to respond to oxidative stress, while pteropods already face severe stress and show limited capacity to deal with further changes. The results suggest that copepods have higher adaptive potential, owing to their stronger vertical migration behaviour and efficient glutathione metabolism, whereas pteropods run the risk of oxidative stress and mortality under high CO_2_ conditions. Our results provide a unique dataset and evidence of stress-inducing mechanisms behind pteropod ocean acidification responses.

## Introduction

The California Current System (CCS) along the west coast of North America from British Columbia to Baja California is a region characterised by seasonal upwelling. Coastal regions of the CCS are currently experiencing pH levels well below those that are expected for the global surface ocean later in this century^1^, and conditions are rapidly changing due to anthropogenic carbon uptake^2^, compromising the health of pelagic calcifiers^3^. The shallow shelf areas off California-Oregon-Washington coasts are expected to become undersaturated with respect to aragonite with increasing frequency over the next few decades^4,5^. In addition, the northeast Pacific was subjected to a marine heatwave event that occurred from late 2013 until the end of 2015^6,7^ overlapping with the strong 2015–16 El Niño^7^. As a result of these climatic phenomena, surface water temperatures in the northeast Pacific reached anomalies of >3 °C above normal^8^ with lasting ecological consequences over the following years.

With ongoing ocean acidification, marine calcifying organisms such as pteropods will be exposed more frequently to low quality pelagic habitat with respect to aragonite saturation state (Ω_arag_), an important parameter affecting the ability for pteropods to form their shells. Ocean warming will add further stress to cool-water pteropods, such as *Limacina helicina*. Pteropod populations have decreased in abundance^9^ and are today considered important bio-indicators for ocean acidification^3^. Pacific pteropods *L. helicina* are essential players in the marine food web, particularly as prey for many commercial fish species^10^.

Copepods, in contrast, are generally considered fairly robust to climate change. Their resilience could partly be linked to stronger vertical migration behaviour, during which they encounter a large gradient of physico-chemical factors^11^. Some species such as *Calanus* spp. diapause at great depths^12^. Crustacean copepods have one of the most lightly calcified cuticles of all crustaceans^13^, and therefore are relatively non-calcifying compared to other taxa, particularly compared to pteropods with aragonitic shells. The species *Calanus pacificus* is an abundant member of the zooplankton community in NE Pacific waters^14^, and forms a key species throughout the CCS^15^. *C. pacificus* is important in food webs, and has declined strongly in abundance during El Niño-related marine heatwaves^15,16^.

Biomarkers have increased in popularity among ecologists^17^, predominantly because of interest in the role of oxidative stress in life-history tradeoffs^18^ and for understanding the mechanisms behind animal behavioural, reproductive, and survival-related performance^19^. Under steady-state conditions, reactive oxygen species (ROS), such as peroxides and superoxide, formed as a by-product of oxygen (Table 1), are scavenged by various antioxidative defence mechanisms^20^. The balance between oxidative stress and antioxidant defences can be perturbed as a consequence of warming, acidification, pollution, hypoxia^21–23^, natural variability^24^, or differential allocation of resources to immunity, reproduction, or growth^19^. When the equilibrium between pro-oxidants and antioxidants is unbalanced, due to high pro-oxidants or insufficient antioxidant activity, this can lead to lower survival^18^. An understanding of cellular responses due to oxidative stress would provide important insight to the stress effects and factors that set limits to species’ tolerance to ocean temperature, oxygen, and inorganic carbon system conditions in the natural oceanic habitat.

**Table 1:** A summary of used biomarkers and their functions in oxidative stress or antioxidant defences.

	Biomarker	Function	Interpretation
GSH: GSSG	Reduced: oxidised glutathione ratio	Assays glutathione redox state	Low ratio or increase in GGSG indicate stress
GP	Glutathione peroxidase	Reduces H_2_O_2_ and lipid peroxides to water and lipid alcohols	↑ indicates more A
GR	Glutathione reductase	Reduces GSSG back to GSH	↑ indicates more A
GST	Glutathione-S-transferase	Catalyses reactions that detoxify harmful compounds	↑ indicates more A
SOD	Superoxide dismutase	Part of enzymatic defence to remove O_2_^−^	↑ indicates more A
CAT	Catalase	Part of enzymatic defence to remove H_2_O_2_	↑ indicates more A
ORAC	Oxygen Reactive Absorbance Capacity	Assesses antioxidant capacity	↑ indicates more A
LPX	Lipid peroxidation	Oxidative degradation of lipids in cell membrane, resulting in cell damage	↑ indicates stress
ETS	Electron Transport System	A biochemical measure of metabolic activity	increases with temperature until a certain °C degree

Biomarker units in methods. A = antioxidant, indicates that biomarker has anti-oxidative function, O_2_^−^ = superoxide radical, H_2_O_2_ = hydrogen peroxide. Modified from Glippa *et al*.^31^.

**Figure 1:**
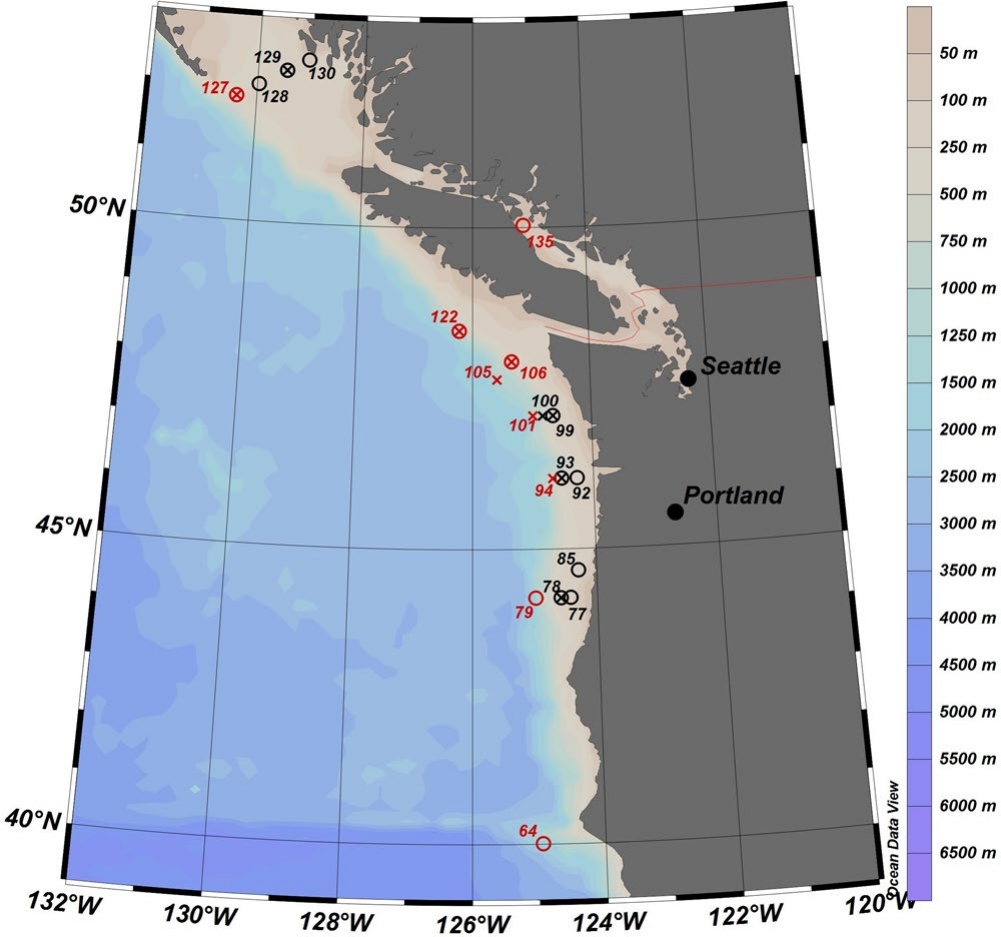
Overview map of the study area showing location and station number of WCOA2016 sampling stations. Copepods and pteropods were sampled for biomarkers at stations represented by empty circles and crosses, respectively. A circle with a cross inside means that both copepods and pteropods were collected at the station. Offshore stations (>200 m water depth) are red, and onshore stations (<200 m) are black. Copepods were collected for ETS (Respiratory Electron Transport System) activity at stations 79, 85, 99, 122, 128, and 129.

The primary objective of this study was to relate physiological condition of field- collected pteropods and copepods (Fig. 1) to oceanographic conditions that are expected to change significantly with climate change, including mainly temperature and carbonate biogeochemistry. By relating oxidative stress to field conditions, we can examine biomarkers measured *in situ* in both taxa over a range of biogeochemical and temperature conditions. To our knowledge, this is the first study to report and compare biomarker outcomes for pteropods and copepods collected over a large gradient of temperature and biogeochemical conditions. Our main hypothesis is that the pelagic pteropod *L. helicina* shows higher oxidative stress and lower antioxidant defences compared with *C. pacificus* copepods.

## Results

**Figure 2:**
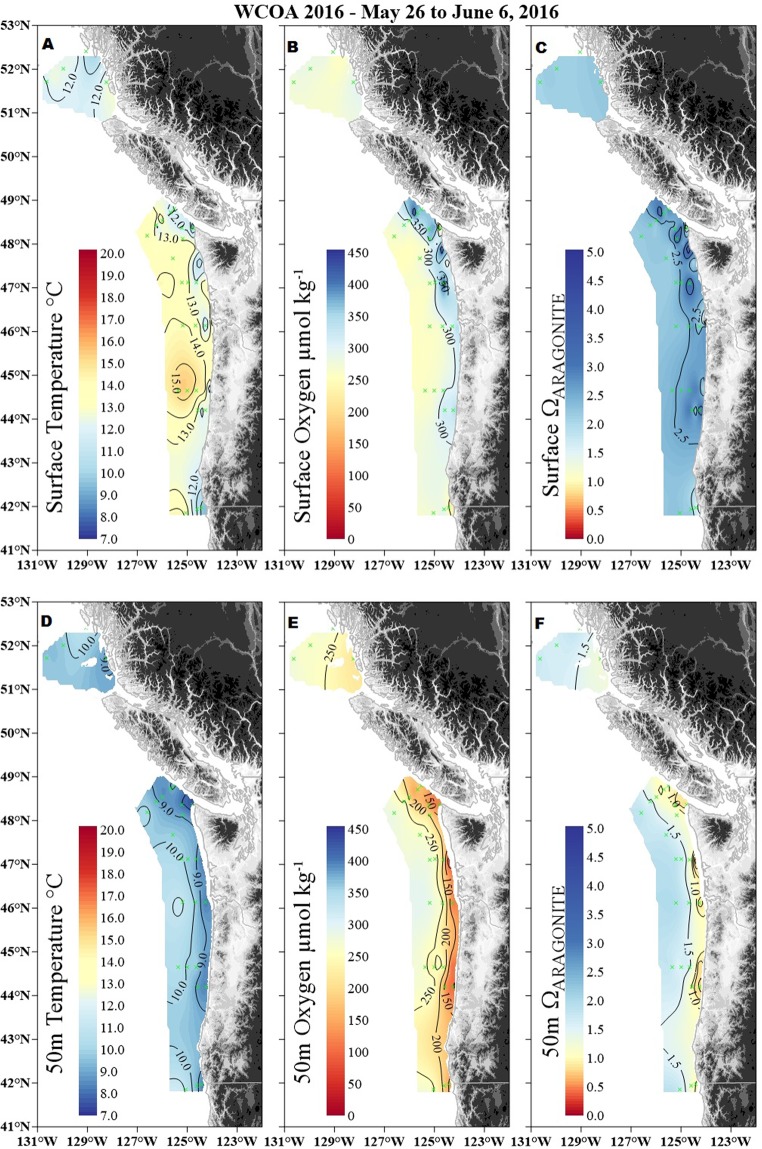
Temperature, oxygen, and aragonite saturation state in the surface waters (**A**–**C**) and at 50 m depth (**D**–**F**) of the Northern California Current System (Oregon-Washington-British Columbia). Carbonate chemistry gradients reflect a range of conditions relevant to ocean acidification, along which biological samples were taken at selected stations represented by green dots. Ω = 1 indicates that carbonate minerals are in equilibrium with the surrounding seawater.

The results from the 2016 West Coast Ocean Acidification (WCOA2016) cruise to the northern California Current System (NCCS, Fig. 1) indicated that seawater property distributions reflected the strong influence of nearshore upwelling in the NCCS where pteropods and copepods were collected. The collective signatures of upwelling and the biogeochemical processes stimulated by macronutrients supplied by upwelling were quite different between the surface and 50 m depth (Fig. 2). At 50 m depth, temperatures, aragonite saturation state, and oxygen concentrations were lower nearshore than offshore, due to upwelling of deeper, colder, oxygen-depleted, and nutrient- and CO_2_-rich waters with low aragonite saturation states. At the surface, this cold water can rapidly exchange heat and gases with the atmosphere while warming, consequently resulting in degassing of CO_2_, uptake of O_2_, and elevating the aragonite saturation state. These changes take place along with the micro-algal uptake of CO_2_ and release of O_2_ in the nutrient-rich, well-lit surface ocean waters. As a result of biological cycling and gas exchange, the average apparent oxygen utilisation observed on the WCOA2016 cruise between 40 and 50°N went from as much as −30 µmol kg^−1^ O_2_ at the surface (0–10 m) to 90 µmol kg^−1^ O_2_ at mid depth (between 45 and 55 m). The highest surface aragonite saturation states were observed in nearshore surface waters where biological drawdown of CO_2_ was intense. Surface temperatures in May and June varied around 12–13 °C in the northern part of the study area, whereas temperatures were slightly higher (13–15 °C) off the Oregon-Washington coasts (Fig. 2). Temperatures were lower at the 50 m depth horizon and varied between 8–11 °C in the study area. Dissolved oxygen concentrations were high in the surface waters varied between 250–450 µmol kg^−1^. At 50 m depth, the dissolved oxygen concentrations were considerably lower, in the range of 100 and 280 µmol kg^−1^. Surface Ω_arag_ ranged from 2.0 to 3.5, but at 50 m depth, Ω_arag_ values were much lower, ranging from 0.5 to 1.5 in nearshore waters and 1.0–2.0 in offshore waters. Farther away from the coast, Ω_arag_ was approximately 1.5 at 50 m depth (Fig. 2).

**Figure 3:**
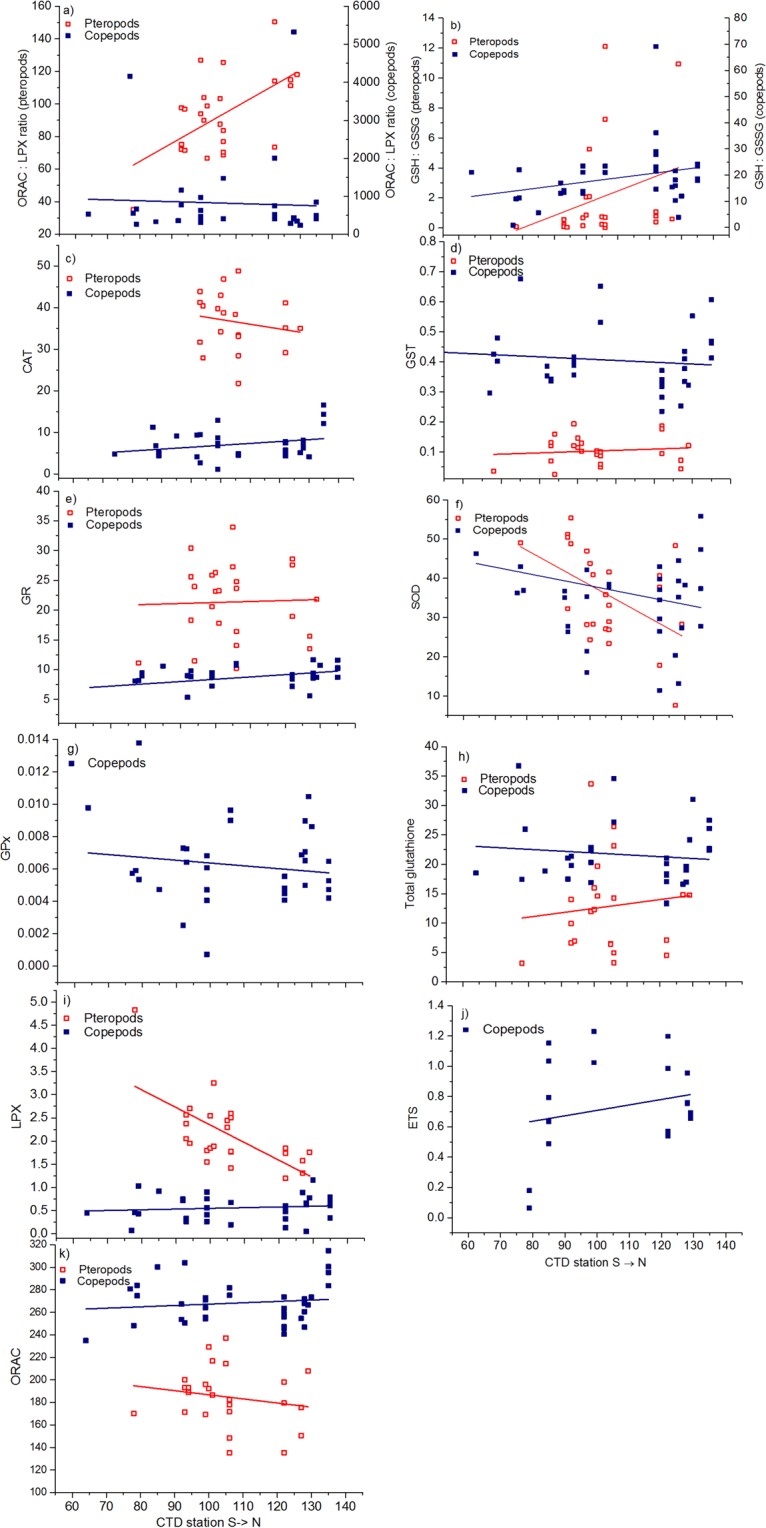
Biomarkers of copepods and pteropods by station (latitude; i.e., south S to north N), graphically presenting species differences analysed by Linear Mixed Models (Table 2). Each point represents a single sample. Units are given as: GPx, GR, GST, and CAT in µmol min^−1^ mg^−1^ protein, SOD as % inhibition, and total glutathione given as µM mg^−1^ protein, LPX as µM cumene-hydroperoxide equivalents mg^−1^ protein, and ETS as µ_1_ O_2_ h^−1^ ind^−1^. Abbreviations as in Table 2.

All biomarkers including both oxidative stress and antioxidant defences, except Superoxide dismutase (SOD), were significantly different between pteropods *L. helicina* and copepods *C. pacificus* (Linear Mixed Model, usually p < 0.001; Fig. 3, Table 2), apart from GP (glutathione peroxidase) and ETS (electron transport system) that were measured only in copepods. Glutathione system (GST) activity, total GSH (glutathione) concentration, Oxygen Radical Absorbance Capacity (ORAC), and reduced to oxidised glutathione (GSH:GSSG) ratio were higher in copepods compared with pteropods. Glutathione reductase (GR) and catalase (CAT) activities and the lipid peroxidation (LPX) were higher, and ORAC:LPX ratio was lower in pteropods than in copepods (Fig. 3). ORAC:LPX and GSH:GSSG ratios (the lower ratios, the more oxidative stress) in pteropods decreased from north to south, while LPX concentration and SOD activity increased towards the south (Fig. 3). For copepods, there were no significant major changes in biomarkers with latitude.

**Table 2:** Linear mixed models comparing responses shown by biomarkers between species *Calanus pacificus* and *Limacina helicina*, and effects by environmental conditions (temperature, and *p*CO_2_ or pH), including significant interactions species × temperature, or species × *p*CO_2_.

Biomarker or ratio	RF variance	factors	Value ± S.E.	df	*t*	*p*
LPX	0.02	species	8.50 ± 3.53	29.36	−2.41	0.022585*
		°C	−0.57 ± 0.41	23.06	−1.38	0.18
		*p*CO_2_	−0.007 ± 0.002	43.54	−4.01	0.000233***
		species × °C	0.64 ± 0.30	27.28	2.11	0.043753*
		species × *p*CO_2_	0.0071 ± 0.0016	42.92	4.43	**6.44 × 10** ^− **5**^ ***
GSH:GSSG	11.79	species	−17.32 ± 2.55	53.70	−6.80	**8.78 × 10** ^− **9**^ ***
		°C	0.94 ± 2.89	18.83	0.33	NS
		pH	9.96 ± 16.55	17.46	0.60	NS
Total GSH	6.81	species	−8.46 ± 1.81	53.72	−4.66	**2.09 × 10** ^− **5**^ ***
		°C	−0.46 ± 2.09	10.98	−0.22	NS
		*p*CO_2_	0.0059 ± 0.0071	9.84	0.83	NS
GR	6.60	species	12.64 ± 1.23	55.49	10.29	**1.75 × 10** ^− **14**^ ***
		°C	1.42 ± 1.64	10.79	0.87	NS
		pH	−0.48 ± 0.59	10.02	−0.05	NS
GST	4.69 × 10^−5^	species	−0.29 ± 0.02	47.06	−13.06	**2 × 10** ^− **16**^ ***
		°C	0.03 ± 0.02	8.67	1.54	NS
		pH	−0.31 ± 0.12	8.36	−250	0.0359*
SOD	0.0	species	1.06 ± 3.35	56.0	0.32	NS
		°C	2.61 ± 3.19	56.0	0.82	NS
		*p*CO_2_	0.03 ± 0.01	56.0	2.36	0.0277*
CAT	1.89 × 10^−13^	species	30.15 ± 1.38	50.0	21.84	**2 × 10** ^− **16**^ ***
		°C	2.36 ± 1.30	50.00	1.82	NS
		pH	−22.27 ± 7.31	50.0	−3.05	0.00369**
ORAC	57.75	species	−83.60 ± 5.34	54.55	−15.64	**2 × 10** ^− **16**^ ***
		°C	20.63 ± 6.06	4.78	3.40	0.0207*
		*p*CO_2_	0.05 ± 0.02	4.36	2.36	NS
ORAC:LPX	5.77 × 10^−9^	species	−727.14 ± 225.71	53.53	−3.22	0.00217**
		°C	−85.66 ± 214.60	53.53	−0.39	NS
		*p*CO_2_	−0.27 ± 0.74	53.53	−0.36	NS
ETS	0.06407	°C	−0.58 ± 0.35	2.80	−1.66	NS
		*p*CO_2_	−0.00075 ± 0.0013	2.52	−0.56	NS

**Figure 4:**
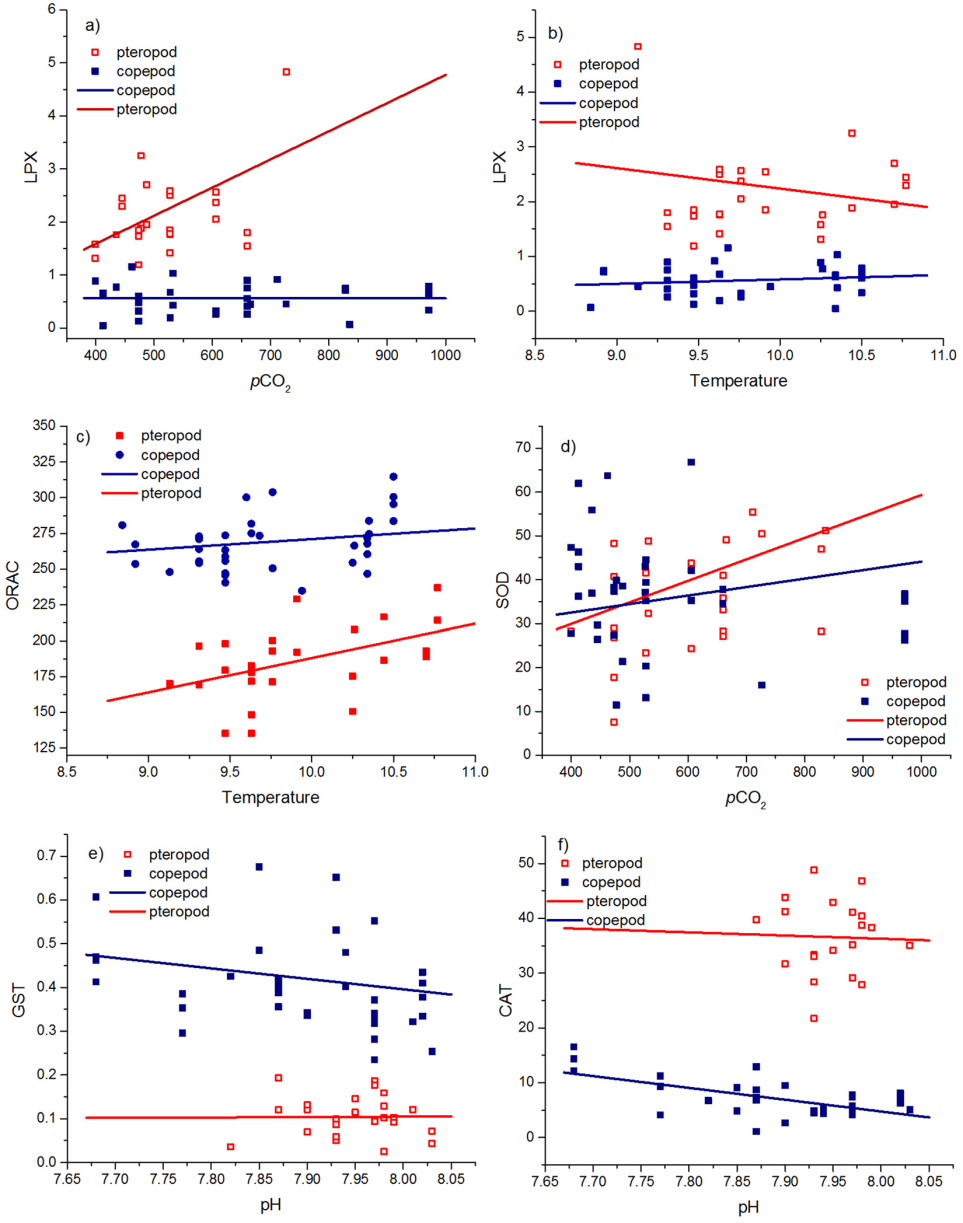
Significant relationships of pteropod and copepod biomarkers in relation to selected environmental variables. (**a**) LPX vs. *p*CO_2_, (**b**) LPX vs. temperature, (**c**) ORAC vs. temperature, (**d**) SOD vs. *p*CO_2_, (**e**) GST vs. pH, and (**f**) CAT vs. pH. The graphs present the mixed model results (Table 2). Abbreviations as in Table 2.

Random factor (RF) was CTD station, i.e., latitude. Significant levels are shown with asterisks (*** < 0.001, ** < 0.01; * < 0.05). See methods for details on statistical analyses, and graphical presentations (Figs 3 and 4). NS = not significant.

LPX = lipid peroxidation; GSH: GSSG = reduced glutathione: oxidized glutathione ratio; Total GSH = total glutathione; GR = glutathione reductase; GST = glutathione-s-transferase; SOD = sodium dismutase; CAT = catalase; ORAC = oxygen reactive absorbance capacity; ORAC: LPX = oxygen reactive absorbance capacity: lipid peroxidation ratio, ETS = electron transport system.

Concerning environmental conditions, the response by species measured as GST and CAT activities was significantly affected by pH (p < 0.05, and p < 0.01, respectively), whereas differences in SOD activity were significantly affected by *p*CO_2_ (p < 0.05). The response by species measured as LPX showed a significant effect by *p*CO_2_ (p < 0.001). In addition, we found that LPX concentrations in species were different due to temperature and *p*CO_2_ (analysed as interactions between species and temperature p < 0.05, or *p*CO_2_ p < 0.0001). *p*CO_2_ concentrations affected also the ORAC activities by the two species (p < 0.01) (Fig. 4).

### Biomarkers and environmental variables

**Figure 5:**
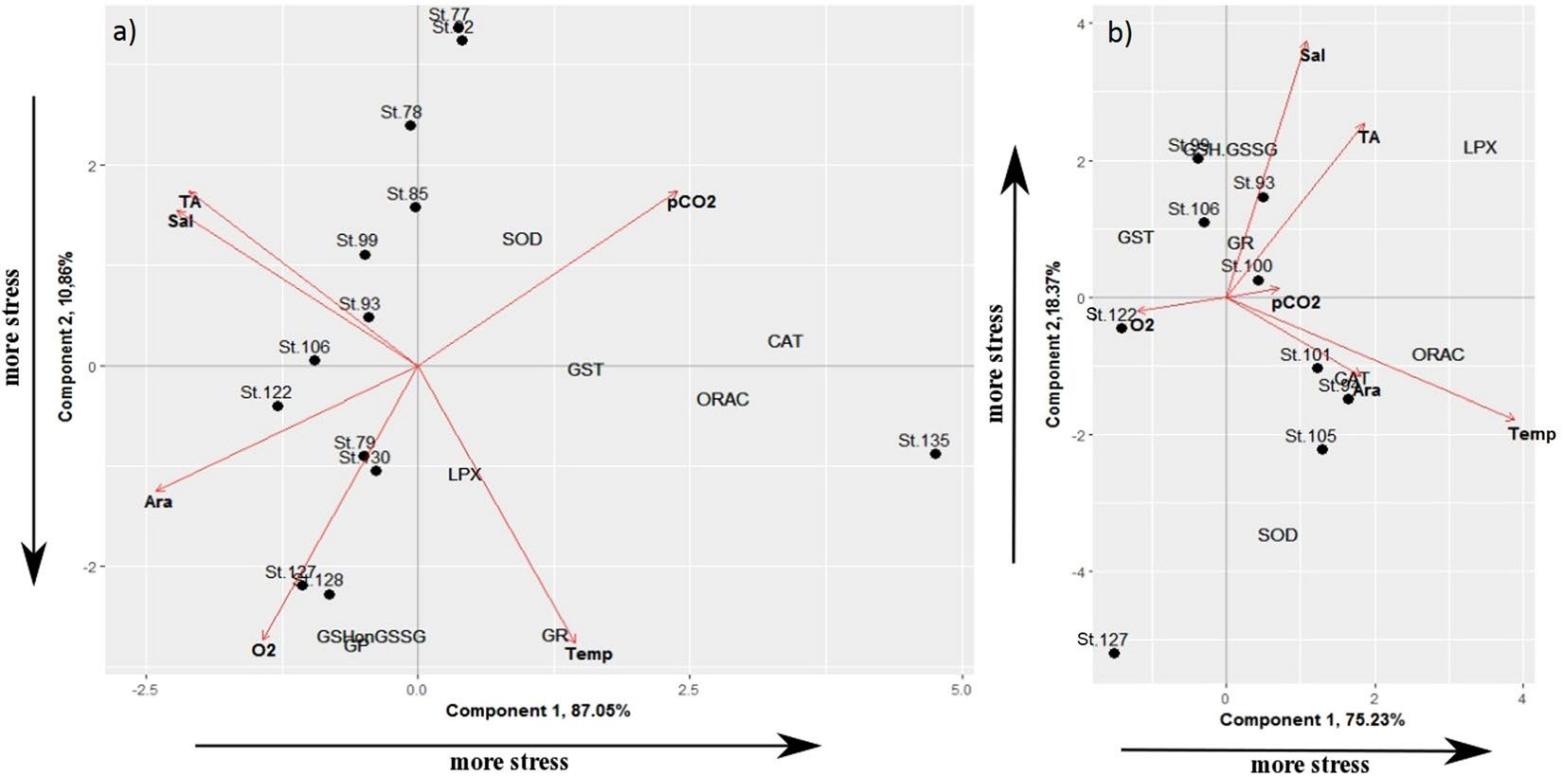
Schematic graphs of Principal Component Analysis (PCA) scores for Components 1 and 2 for copepods *Calanus pacificus* (**a**), and for pteropods *Limacina helicina* (**b**). The corresponding loading plots (biomarker variables and environmental variables) are superimposed on the scores plot. Biomarker abbreviations as in Table 3. Aragonite saturation state (Ara), Temperature (Temp), Salinity (Sal), Dissolved oxygen concentrations (O_2_), partial pressure of Carbon dioxide in seawater (*p*CO_2_), and Total Alkalinity (TA). Environmental data were integrated over the upper 100 m.

In the copepod principal component analysis (PCA), GST, ORAC, and CAT were positively associated with Principal Component 1 (PC1), with *p*CO_2_, aragonite saturation, salinity and total alkalinity (TA) as main environmental drivers. PC2 was positively associated with SOD and negatively associated with GR, LPX, GSH:GSSG ratio and glutathione peroxidase (GP), with temperature and oxygen as the significant environmental variables. Of the total observed variation, 97.9% was explained by the first two principal components, 87.0% by PC1 and 10.9% by PC2 (Fig. 5a, Table 3a).

**Table 3:** Component matrix for Fig. 5, copepods (A), and pteropods (B).

(A) copepods	Component			Component	
	1	2		1	2
GST	**0.310**	−0.006	Temp	0.288	**−0.552**
GR	0.253	**−0.534**	O_2_	−0.286	**−0.545**
SOD	0.193	**0.256**	Ω_A_	**−0.483**	**−**0.249
ORAC	**0.563**	**−**0.064	*p*CO_2_	**0.477**	0.347
LPX	0.087	**−0.213**	TA	**−0.422**	0.347
CAT	**0.678**	0.052	Sal	**−0.443**	0.309
GSH:GSSG	**−**0.085	**−0.537**			
GP	**−**0.111	**−0.555**			
**(B) pteropods**					
GST	**−0.246**	0.177	Temp	**0.779**	**−**0.359
GR	0.038	**0.161**	O_2_	**−0.241**	**−**0.041
SOD	0.142	**−0.691**	Ω_A_	**0.363**	**−**0.230
ORAC	**0.574**	**−**0.163	*p*CO_2_	**0.142**	0.025
LPX	**0.686**	0.442	TA	0.371	**0.507**
CAT	**0.342**	**−**0.234	Sal	0.216	**0.748**
GSH:GSSG	0.014	**0.434**			

Biomarker abbreviations (GST, GR, SOD, ORAC, LPX, CAT, GSH:GSSG, GP) as in Table 1. Environmental abbreviations are as follows: Temperature (Temp), Dissolved oxygen concentrations (O_2_), Aragonite saturation state (Ω_A_), partial pressure of Carbon dioxide in seawater (*p*CO_2_), Total Alkalinity (TA), Salinity (Sal). Loadings highlighted in bold represent the highest absolute value of the parameter when considering different principal components.

In the PCA using pteropod data, ORAC, LPX, and CAT were positively associated with PC1, whereas GST had an inverse relationship with these variables. PC2 was positively associated with GR and GSH:GSSG ratio, and negatively associated with SOD. For pteropods, temperature was the most important environmental factor, and aragonite saturation Ω_arag_ as second most important factor in the PCA. The two main components explained in total > 90% of the observed variance in the PCA: 75.2% by PC1 and 18.4% by PC2 (Fig. 5b, Table 3b).

## Discussion

We present a unique dataset as first evidence to demonstrate that biomarkers measured in copepods and pteropods in the Northern California Current System show strong, yet differential, relationships with temperature and pH/*p*CO_2_. Our results clearly show higher oxidative damage (elevated LPX and decreased GSH:GSSG ratio) in pelagic pteropods compared to the copepods, whereas for most antioxidants, especially the glutathione system (GST and total glutathione), copepods have higher activities.

The ORAC:LPX ratio indicates oxidative stress, with a low ratio expressing a higher level of oxidative stress^25^, and the ratio was approximately one tenth as high in *Limacina* pteropods as in *Calanus* copepods (Fig. 3a). The ORAC:LPX ratio of pteropods decreased towards the south, and despite the ratio did not have a significant relationship with environmental conditions, ORAC was significantly related to temperature. There was also interactions between species and temperature or *p*CO_2_ measured as LPX, showing that pteropods responded more strongly to thermal stress and increasing *p*CO_2_ (Fig. 4), of which the latter was observed to be as high as 725 µatm off the Oregon coast. Also, pH, oxygen and temperature were significant factors affecting antioxidant defences and oxidative stress levels (Figs 4 and 5; Tables 2 and 3), showing that several factors combined are stressful for pteropods. In the PCA, temperature, in addition to Ω_arag_, was by far the most important environmental factor influencing pteropod stress levels. These results suggest harmful changes on a cellular level in pteropods are already occurring at some locations in the NCCS. Low Ω_arag_ combined with higher temperatures could cause the cumulative effects observed in pteropods^26^, and result in elevated lipid peroxidation and antioxidant enzymes^27–29^. Vehmaa *et al*.^25^ reported a decreasing ORAC:LPX ratio as a response to manipulated warming and pH in *Acartia* copepods. Baselines of biomarkers measured in copepods can be difficult to rely on, as spatio-temporal conditions vary greatly among field studies.

Accounting for this, reported LPX concentration ranges from previous copepod studies are 0.1–21 µM cumene-hydroperoxide equivalents mg^−1^ in *Limnocalanus macrurus*^30^, 29–53 in *Acartia* sp.^31^, and 0.3–3.3 µM cumene-hydroperoxide equivalents mg^−1^ in *Calanus finmarchicus* (exposed to severe manipulated ocean acidification, unpubl. data). In the current study *Calanus* spp. LPX concentrations were low, around 0.5 µM cumene-hydroperoxide equivalents mg^−1^ (cf. Fig. 3), suggesting that the copepods can defend themselves against external stress factors at the cellular level, and keep the balance between ROS and antioxidant defences stable.

Biomarkers (CAT, LPX, SOD, GST) that showed a significant relationship with pH or *p*CO_2_ showed the most extreme values in samples collected in the region of regularly occurring upwelling of CO_2_-rich waters off the Washington-Oregon-California coast^1,4^. Upwelling exposes organisms to corrosive waters that are depleted in carbonate ion, a central component for CaCO_3_ shell building^3,26,32^. If pteropod habitat is undersaturated with respect to aragonite (Ω_arag_ ≤ 1), it will lead to shell thinning and dissolution, which increases vulnerability to predation and imposes increased energetic costs^33,34^, especially when undersaturation stress is interacting with other *in situ* stressors, such as thermal stress and hypoxia^35^. The observed pteropod LPX content also showed a relationship with oxygen concentrations in the PCA (Fig. 5b), which would be expected as pteropods are sensitive to hypoxia, in combination with other stressors^36^. The energetic cost for pteropods to cope with acidified conditions has not been determined^37^, but Lischka & Riebesell^37^ suggest that decreased growth causes decreased activity and metabolism, leading to decreased internal lipids, slower calcification, and discontinued development of gonads.

The GSH:GSSG ratio, which is lower for organisms experiencing oxidative stress, decreased in pteropods with decreasing latitude, suggesting that the environmental conditions in the south are more harmful for them than in the north. As GSH:GSSG ratio decreased in pteropods (Fig. 3), GR should increase. As the function of GR is to reduce GSSG to GSH^30^, and thereby elevate the GSH:GSSG ratio, this suggests the enhanced stress in the southern NCCS is exceeding the pteropods’ ability to cope by increasing their GR activity. In mammals, a cell in resting phase has a GSH:GSSG ratio exceeding 100:1, whereas a cell suffering from severe oxidative stress has a ratio ~10:1, or 1:1^30,38^. A low GSH:GSSG ratio indicates cellular toxicity and is a biomarker that works across different functional groups, from plankton to mammals^30,38^. The consequences of a low GSH:GSSG ratio (Figs 3 and 5), in combination with low antioxidant capacity, has so far not been investigated considering pteropod life cycle and mortality risk, but is the focus of our recent efforts^35^. Two thirds of the pteropod GSH:GSSG ratio samples were <1 in the current paper, suggesting serious oxidative stress^30,38^.

Considering differences in habitat use of pteropods vs. copepods as a potential explanation for their differences in biomarkers, *Calanus pacificus* and *L. helicina* seem to perform diel vertical migration (DVM) across fairly similar depth ranges in the California Current (~100 m)^39–42^, although relatively limited data on pteropod DVM are available to characterise the depth they regularly reach. A potentially important difference is that *Calanus* spp. diapause at depths > 1000 m^12^ whereas *Limacina* does not diapause. Lewis *et al*.^11^ observed that adult *Calanus* sp. in the Arctic, which migrate daily across a >140 μatm gradient of *p*CO_2_ in their natural habitat, exhibit little response to high CO_2_ in the laboratory, indicating a potential resilience to high CO_2_ conferred by their daily exposure. Thus, perhaps via carry-over effects (i.e., effects of previous experience that persist through the life cycle^43^), copepods that are exposed to a large range of physico-chemical conditions that elicit production of ROS over their life cycle may have higher base levels of antioxidants for efficient elimination of ROS than do pteropods.

In addition, copepods have an efficient glutathione metabolism^44^ (see Fig. 5), which responds rapidly to changing hydrography^30,31^. *Calanus* showed higher antioxidant defences (GSTs, total GSH) than pteropods, indicating a larger capacity for ROS scavenging^45^. This is also supported by our observations of elevated ORAC in copepods^30^, detected in warmer conditions and in low pH waters during a laboratory study. Detoxification is a fundamental defence mechanism, which allows the organism to survive or even thrive despite oxidative stress, with the GST superfamily, a set of enzymes involved in the detoxification process, being involved in this^44^. Previous work suggests that copepods are more sensitive to warming than to acidification^25,46,47^, and shown by using biomarkers in Vehmaa *et al*.^25^. Also, a review by Havenhand^48^ suggests that copepods will likely be resilient to near-future OA.

Catalase (CAT) is an antioxidant enzyme that directly converts hydrogen peroxide (H_2_O_2_), a harmful substance for the cell, to oxygen and water. CAT activities were much higher (33–43 µmol min^−1^ mg^−1^) in pteropods than in copepods (4–14 µmol min^−1^ mg^−1^), suggesting much more ROS in pteropods to be detoxified by CAT. In contrast, CAT activity increased in copepods when pH decreased, whereas this was not the case in pteropods. This suggests that copepods have the capacity to respond to changes in carbonate chemistry, encountered for example during vertical migration, by increasing their antioxidant capacity^49^, whereas pteropods show no indication of further increasing their response to additional stress, suggesting combined thermal and acidification stress may push their antioxidative activity to the limits. While SOD was the only biomarker that was not significantly different between copepods and pteropods, SOD activity increased towards the south in both species, again pointing to more severe conditions requiring a response of the antioxidant defence system.

Pinheiro & Oliveira^50^ showed that crustacean CAT and SOD that are in the first line of antioxidant defence against H_2_O_2_ and superoxide, respectively, respond sensitively to changes in environmental conditions, such as thermal stress. Similarly to CAT, glutathione peroxidase (GP) converts H_2_O_2_ into oxygen and water. Vuori *et al*.^30^ showed that GP in Baltic *Limnocalanus* copepods (0.01–0.29 µmol min^−1^ mg^−1^) was inversely related to lipid peroxidation, which, in turn, suggests the presence of lingering ROS. GP activities in *Calanus* in the CCS were 0.003–0.01 µmol min^−1^ mg^−1^, not revealing any acute stress in copepods. In the PCA of our data, many of the antioxidant biomarkers (Table 3, Fig. 5) were strongly associated with each other, but not negatively associated with LPX, as one would expect cf.^30^. The PCA emphasises the differences in the stress responses of copepods and pteropods with respect to various environmental stressors. LPX increased at higher temperature and decreased at higher salinity in copepods, whereas in pteropods, LPX showed a stronger relationship with higher TA and salinity. In copepods the GP activity and GSH:GSSG ratio were higher in high O_2_ and low *p*CO_2_^,^ whereas for pteropods there does not seem to be such a good relationship. Temperature seems to affect glutathione recycling in copepods, while in pteropods the increase in ORAC and CAT indicates that other antioxidant routes are activated instead of the glutathione system.

Since all pteropods were collected from deeply towed nets whereas some copepods were collected from relatively short near-surface tows, differences due to the effects of sampling stress also cannot be ruled out. Nets were pulled slowly, but deep tows were pulled for fairly long time periods, and this may have caused increases in enzymes that handle ROS directly and that have a fairly rapid turnover. These procedures may have caused higher variability in the results.

In the present study, the ORAC increased only in pteropods with elevated temperature. Several papers suggest that both copepods and pteropods are sensitive to ocean warming^37,47^. For example, in copepods, egg production and hatching can decrease with increasing temperature^25,51^, whereas in pteropods (*Limacina helicina*, *L*. *retroversa*), warming can amplify the effects of *p*CO_2_ on shell degradation and increase respiration and mortality^33^.

ETS reflects maximum potential respiratory rates, and was measured in *Calanus* copepods at five different stations in the present work. The measured rates of 0.06–1.23 µL O_2_ h^−1^ ind.^−1^ compare well with rates of 0.14–4.46 µL O_2_ h^−1^ ind.^−1^ reported for *Calanoides* in the northern Benguela upwelling system^52^. ETS did not show a relationship with *in situ* temperature, which was opposite to what we had expected^53^. This result could be due to the fact that the *in situ* temperature we considered was integrated over the upper 100 m of the water column, but the time the copepods spend over those depths is not distributed evenly as they spend the day at depth and are at the surface feeding during the relatively short nights.

Another result of depth integrating temperature is that the range among stations was only 1 °C, which is much less than they experience during DVM. Many other factors can also influence ETS, such as food availability. Further, differences in body mass, depth of occurrence, and general animal activity could explain up to 90% of the variance in respiratory rates^52^. *Calanus* is a fast-moving copepod and its fairly high ETS level could partly be explained by general activity.

To conclude, copepods and pteropods responded differently to environmental conditions, such as warming, hypoxia, and low pH. Copepods showed responses in measured biomarkers indicative of stress in relation to temperature, *p*CO_2_, pH, and O_2_, by increasing their antioxidant defence enzymes that scavenge harmful reactive oxygen species. Pteropods also responded to temperature, Ω_arag,_ and pH and were severely affected by oxidative stress that can lead to cell damage and death. Pörtner & Farrell^54^ demonstrated that synergistic stressors like ocean acidification and hypoxia narrow thermal windows according to species-specific sensitivities, i.e., climate change will differentially favour species with wide thermal windows, short generation times, and a range of genotypes among its populations. Our results suggest that copepods are more tolerant than pteropods to current conditions, which can be related to their vertical migration behaviour^11^. Copepods also have efficient glutathione metabolism, allowing fast responses to changing ROS conditions^30,31^. Our data suggest that pteropods are sensitive to changes to temperature, Ω_arag,_ pH, and oxygen. Further studies are needed to investigate biomarkers of these two groups that both form major prey for commercial fish species.

## Methods

### Sampling

This study took place in the northern California Current System (NCCS) during the fifth NOAA West Coast Ocean Acidification cruise (WCOA2016, https://www.nodc.noaa.gov/oceanacidification/data/0169412.xml), which was conducted throughout the entire CCS in May–June 2016. The samples were collected on-board the NOAA Ship *Ronald H. Brown*, from which a total of 19 stations were sampled from 39.5 to 53°N, 124 to 131°W (Fig. 1, Supplementary Table 1). Copepod *Calanus pacificus* and the pteropod *Limacina helicina* were targeted. Organisms were collected mainly using a Bongo net (⌀ 60 cm, 333 µm mesh) with a non-filtering cod end, towed obliquely over the upper 100 m at a speed of 2 knots, usually for 25–40 min. Additional copepod samples were collected opportunistically from surface tows using a 0.5 × 1.0 m, 333 µm mesh neuston net towed at the surface (0–2 m) for 15 min. The two nets were used to maximise the catch. After each net tow, the animals were transferred to 20L coolers filled with seawater from the subsurface, and stored at ambient temperature. Other organisms (i.e., fish or crab larvae, and jellyfish) were removed from the cooler to avoid stress to target animals. We sorted both taxa on ice under binocular microscopes, each individual picked with a pipette to a petri dish and transferred with tweezers to a 2 mL plastic Eppendorf tube, then flash-frozen in liquid nitrogen. *Limacina helicina* and *Calanus pacificus* (Number of samples = 1–7 per taxonomic group) were picked per station depending on availability. Each copepod sample consisted of ~15 adult females (females selected as they reproduce), and each pteropod sample consisted of ~20 individuals. In total, 60 samples were collected (35 copepod, 25 pteropod samples), and they were transported by air on dry ice to Finland, and stored in −80 °C until biomarker analysis was conducted.

### Chemical measurements

As part of the WCOA2016 cruise, which included 17 cross-shelf transects, we obtained measurements of conductivity-temperature-depth (CTD) and oxygen from a Sea-Bird SBE 911 plus CTD system. At each station, water samples were collected in modified Niskin-type bottles, poisoned with HgCl_2_ and analysed on-board the ship for dissolved inorganic carbon (DIC), total alkalinity (TA), pH, chlorophyll *a* (chla), and oxygen using the methods described in Alin *et al*.^55^). The ship-based DIC and TA data are both precise and accurate to within 2 µmol kg^−1^.

### Biomarkers

In the present study, we used lipid peroxidation LPX and total GSH (glutathione) concentrations; Catalase CAT, Superoxide dismutase SOD, Glutathione S-transferase GST, Glutathione peroxidase GP, Glutathione reductase GR, ETS activities, Oxygen Radical Absorbance Capacity ORAC; and reduced to oxidised glutathione GSH:GSSG ratio (Table 1), as biomarkers of stress responses in copepods and pteropods collected along the NCCS. To prevent oxidative stress and damage to biomolecules, such as LPX, organisms use antioxidants to counteract ROS (reactive oxygen species). SOD catalyses the dismutation of superoxide O^•−2^ to oxygen and H_2_O_2_, which is further catalysed by CAT to ground state O_2_ and water by GP. GSH is one of the most important and active non- enzymatic antioxidant defence in biological systems and it acts as cofactor for GP, or react with ROS independently. In the reaction with GP, GSH is oxidised, forming GSSG, which can be reduced by GR. The ratio between the reduced and oxidised forms of glutathione has been considered an important indicator of the redox status of cells. GSH is also part of xenobiotic metabolism, by acting as a conjugant in a reaction catalysed by GST enzymes. Some GSTs can catalyse the reaction of organic peroxides with GSH, thus preventing LPX^56^.

Zooplankton samples were homogenised on ice in 150 µL (*Calanus pacificus*) or 100 µL (*Limacina helicina*) of 0.1 M K_2_HPO_4_ + 0.15 M KCl buffe^r^ (pH 7.4) using a Tissue Lyser II bead mill (Qiagen). 25 µL of raw homogenate was directly frozen in liquid nitrogen and stored at −80 °C for lipid peroxide determination (LPX). Then, the homogenate was centrifuged at 10,000 g for 15 min at 4 °C and the resulting supernatant was divided into aliquots for GST, GR, GP, CAT and SOD enzyme activity determination, ORAC assay and for glutathione sample preparation (GSH, GGSG). The glutathione sample was deproteinised by adding 5% sulfosalicylic acid (SSA). The sample was incubated on ice for 10 min and centrifuged for 10 min at 10,000 g at 4 °C. The supernatant was divided into two different tubes for reduced (GSH) and oxidised glutathione (GSSG) and 33 mM M_2_VP (1-methyl-2-vinylpyridinium trifluoromethanesulfonate, Sigma Chemicals) in 0.1 M HCl that is a scavenger of GSH, was added to the GSSG sample. The sample homogenate aliquots and glutathione samples were frozen in liquid nitrogen and stored at −80 °C until further analysis.

GSH and GSSG were analysed with Glutathione 384-well plate Fluorescent Detection Kit (Arbor Assays) and intracellular soluble antioxidant capacity with OxiSelectTM Oxygen Radical Antioxidant Capacity (ORAC) Activity Assay (Cell Biolabs), following the manufacturers’ instructions, except for adjusting the reaction volumes for 384-well plate when needed. GST, GR, CAT, SOD, and LPX were determined as described in Vuori & Kanerva^57–61^. GP was only measured for copepods according to the protocol of Vuori & Kanerva^62^.

The enzyme activities, lipid hydroperoxides, and total GSH were normalised to the sample protein content, which was determined with PierceTM BCA Protein Assay (Thermo Scientific) with bovine serum albumin (Sigma) as the standard. The ORAC Assay is a tool for measuring the general antioxidant capacity of samples. Antioxidant systems of the sample work to block the peroxyl radical oxidation (caused by addition of free radical initiator) of the fluorescent probe until antioxidant activity in the sample is depleted. All samples, standards and blanks were analysed in triplicate. For all assays in this study, the mean coefficient of variation (CV%) of the technical replicates ranged between 2.77 and 7.30% (copepods) and between 3.43 and 5.33% (pteropods).

For Respiratory Electron Transport System (ETS), adult female Calanus copepods were identified to genus under the microscope and 7–8 individuals per sample were flash frozen on liquid nitrogen and stored at −70 °C. The ETS activity was measured according to Owens & King^63^, modified by Gómez *et al*.^64^, and adapted for a 96-well plate. Briefly, samples were homogenised in a Teflon glass grinder at 2 °C for 1.5 min in 20 mM Tris Buffer (pH 7.8), then centrifuged at 1503 × g for 10 min at 2 °C. ETS activity was measured via INT (2-(p-iodophenyl)-3-(p-nitrophenyl)-5-phenyl tetrazolium chloride) reduction to formazan by the change in absorbance, measured kinetically at 490 nm with spectrophotometer (SpectraMax M2, Molecular Devices). For each assay, 30 µL of the homogenate was added to 90 µL substrate solution (1.7 mM NADH and 0.25 mM NADPH dissolved in phosphate buffer), and the reaction was initiated by adding 30 µL INT (0.2%, pH 8.5). Blank measurements were taken using phosphate buffer (0.1 M phosphate buffer pH 8.5, 0.2% v/v Triton x-100, 0.15% w/v polyvinylpyrrolidone, 75 µM MgSO_4_) without added substrates. Assays and blanks were measured in triplicate at 24 °C and corrected to *in situ* temperatures (depth integrated from 0–100 m) using the Arrhenius equation with an activation energy of 15 kcal/mol^65^; potential respiration was calculated according to Packard & Christensen^66^.

### Statistical analyses

We used linear mixed models (LMMs), based on restricted maximum likelihood estimation, for comparing biomarker levels between species and for analysing effects of environmental conditions. Biomarker levels were treated as the response variable, species as the fixed-effect (categorical) factor and environmental conditions (integrated over the upper 100 m) as fixed-effect co-variates, and CTD (i.e., conductivity-temperature-depth) station was a random effect. Multicollinearity was evidenced by correlations between environmental variables exceeding 0.7; such variables were not included in the same model. Selection between collinear variables was based on comparing the Akaike’s information criterion (AIC) of single-variable candidate models explaining biomarker levels, with lower values of AIC being preferred^67^. These candidate models were fitted using maximum likelihood to allow AIC-based comparison of models with different fixed effects. The variable with the lowest AIC was therefore selected for the final model (aragonite was not considered, as it is important only for one of the study species, *L. helicina*). To facilitate models convergence, only two co-variates were included per model. In cases of unfavourably low ratios of observations to variables, indicating overfitting, we omitted two-way interactions from the model. For LMMs, the package ‘*lmerTest*’ was used in the free software R^68^, version 3.4.3 (www.R-project.org). pH was transformed to [H+] prior to analyses, as pH is a logarithmic unit.

In order to visualise the associations among measured antioxidant defence and oxidative stress variables and environmental data, a three-table ordination method, the species traits analysis (=RLQ analysis)^69^, was used. This involves the construction of three data tables: a table with variables describing the environmental conditions at the stations (R), a table containing traits (e.g., biomarkers) of the species (Q), and a table with presence-absence values for species at a series of stations (L). First, a principal component analysis (PCA) was performed for biomarkers and the environmental data, then a correspondence analysis on the sample groups (i.e., CTD stations). These three analyses were passed to the RLQ function of ‘*ade4*’ package in R, version 3.4.3 (www.R-project.org).

## Supplementary information

Supplementary table S1
