# Supplementary material for: Eco-physiological responses of copepods and pteropods to ocean warming and acidification
Source: Sci Rep. 2019 Mar 18;9:4748. doi: 10.1038/s41598-019-41213-1 (PMC6426838; doi:10.1038/s41598-019-41213-1)
Supplement: Supplementary file 1 — Supplementary table S1 [file 41598_2019_41213_MOESM1_ESM.pdf]

Eco-physiological responses of copepods and pteropods to ocean warming and acidification

J. Engström-Öst, O. Glippa, R. A. Feely, M. Kanerva, J. E. Keister, S. R. Alin, B. R. Carter, A. K. McLaskey, K. A. Vuori, N. Bednaršek

Supplementary Table S1. Number of onshore and offshore biomarker samples collected at CTD stations 64-135.

Station	Samples	
	Calanus	*Limacina*
64	1	
77	1	
78	1	1
79	2	
85	1	
92	2	
93	2	3
94		2
99	5	2
100		2
101		2
105		2
106	2	5
122	7	3
127	1	2
128	4	
129	1	1
130	1	
135	4	

(white)	offshore>200m
(grey)	onshore≤ 200 m
